# Harnessing RNA therapeutics: novel approaches and emerging strategies for cardiovascular disease management

**DOI:** 10.3389/fcvm.2025.1546515

**Published:** 2025-03-20

**Authors:** Wang Dui, Zhao Xiaobin, Zhang Haifeng, Dang Lijuan, Huang Wenhui, Zhang Zhengfeng, Song Jinling

**Affiliations:** ^1^ Department of Cardiovascular Rehabilitation, The Third Affiliated Hospital of Gansu University of Traditional Chinese Medicine, Baiyin, China; ^2^ Department of Endocrinology, The Third Affiliated Hospital of Gansu University of Traditional Chinese Medicine, Baiyin, China; ^3^ Cardiovascular Department, The Third Affiliated Hospital of Gansu University of Traditional Chinese Medicine, Baiyin, China

**Keywords:** cardiovascular diseases, RNA therapeutics, mRNA, siRNA, miRNA, RNA aptamers, RNA delivery systems

## Abstract

RNA therapeutics are emerging as a promising approach for cardiovascular diseases (CVDs) management, offering targeted gene regulation through modalities like mRNA, siRNA, and miRNA. In recent years, researchers have conducted a lot of research on the application of RNA therapeutics technology in the treatment of CVDs. Despite hurdles in off-target effects and immune responses, the clinical trial outcomes are encouraging. This review synthesizes the current progress in RNA therapeutics for CVDs, examining their mechanisms, advantages, and challenges in delivery and safety. We highlight the potential of personalized medicine, combination artificial intelligence (AI) and bioinformatics in advancing RNA therapeutics. The future of RNA therapeutics in CVDs is poised for significant impact, necessitating continued research and interdisciplinary collaboration to optimize these treatments and ensure patient safety and efficacy.

## 1 Introduction

Cardiovascular diseases (CVDs) encompass a range of disorders affecting the heart and blood vessels, including coronary artery disease, stroke, heart failure, and hypertension (1). According to the World Health Organization, CVDs are the leading cause of death globally, claiming approximately 17.9 million lives each year, which accounts for 32% of all global deaths (2). Notably, ischemic heart disease and stroke represent the most prevalent forms of CVDs, with the Global Burden of Disease Study reporting that ischemic heart disease alone accounted for 8.9 million deaths in 2019 (3). The prevalence of CVDs is expected to rise, particularly in low- and middle-income countries due to aging populations, lifestyle changes, and increasing rates of obesity and diabetes (4).

The global burden of CVDs goes beyond just mortality; it also has a considerable impact on healthcare systems and economies around the world (5). According to the American Heart Association, direct medical costs and indirect productivity losses attributed to CVDs exceeded $200 billion annually in the United States alone (6). Disparities in CVDs outcomes are evident, with variations in incidence and mortality rates among different populations attributed to factors such as access to healthcare, socioeconomic status, and lifestyle choices (7). For instance, studies demonstrate that individuals with low socioeconomic status are at a heightened risk for CVDs, with mortality rates up to three times higher compared to their higher-income counterparts (8). This variability highlights the need for targeted interventions and public health strategies to effectively manage and mitigate CVDs risk, particularly in disadvantaged populations.

RNA therapeutics have emerged as a groundbreaking approach in modern medicine, leveraging the potential of RNA molecules to modulate gene expression and protein synthesis (9). These therapeutics encompass a variety of modalities, including small interfering RNA (siRNA), messenger RNA (mRNA), microRNA (miRNA), and antisense oligonucleotides (ASOs) (9). The success of mRNA vaccines against COVID-19, such as those developed by Pfizer-BioNTech and Moderna, has demonstrated the power of RNA technology to elicit robust immune responses and has ushered in a new era of rapid vaccine development (10). Furthermore, RNA therapeutics can be designed to target specific pathways involved in various diseases, providing a tailored treatment method that offers advantages over traditional small-molecule drugs, particularly in terms of specificity and reduced off-target effects (11).

In the context of CVDs, preclinical trials have shown promising results. For instance, siRNA targeting PCSK9 has been effective in reducing LDL cholesterol levels in animal models (12). Similarly, mRNA encoding for vascular endothelial growth factor (VEGF) has demonstrated potential in promoting angiogenesis and improving myocardial perfusion in preclinical studies (13, 14). The therapeutic use of miRNA involves either inhibiting the activity of pathologically upregulated miRNAs or replacing downregulated protective miRNAs (15). For example, let-7 family inhibition has demonstrated beneficial effects on cardiac remodeling following injury (16). ASOs have also shown potential in preclinical trials, with studies demonstrating their ability to target specific genes involved in lipid metabolism and inflammatory pathways (17).

The objective of this review is to comprehensively summarize the current state of RNA-based therapies in the management of CVDs, highlighting recent advancements, ongoing clinical trials, and emerging strategies. We aim to critically evaluate the efficacy and safety of these therapies, discussing variability in study outcomes and the underlying factors that contribute to these differences. By synthesizing and contextualizing these findings, this review will provide valuable insights into the potential of RNA therapeutics as transformative approaches in CVDs management, paving the way for future research and clinical application.

## 2 RNA therapeutics

**Figure 1:**
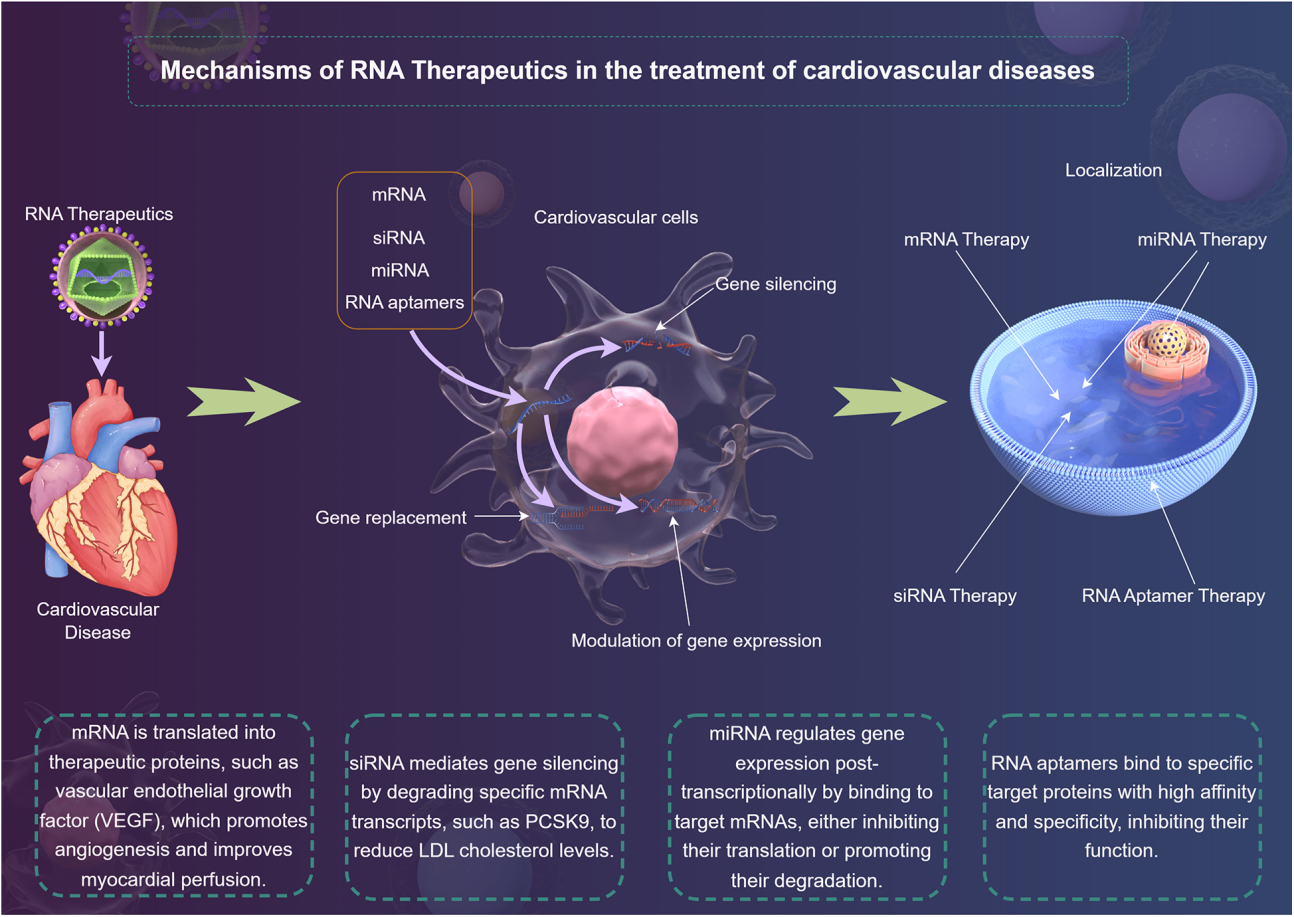
The mechanism of RNA therapy for cardiovascular disease was demonstrated by Figdraw.

In recent years, RNA therapeutics have emerged as a promising modality in the treatment of CVDs (18). With the capability to directly modulate gene expression and cellular processes, RNA-based approaches provide novel avenues for intervention in the complex cellular networks that underlie cardiovascular pathologies (19). The main types of RNA therapeutics include mRNA (20), siRNA (21), microRNA (miRNA) (15), and RNA aptamers (22) (Figure 1). They act on diseases through mechanisms of action such as gene silencing (23), gene replacement (24) and modulation of gene expression (25), and have advantages in the treatment of CVDs (26).

### 2.1 Types of RNA therapeutics

mRNA serves as a template for protein synthesis and has garnered significant attention in the development of RNA therapeutics (20). The advent of lipid nanoparticles (LNPs) technology has enabled efficient delivery of mRNA vaccines, as witnessed during the COVID-19 pandemic (20). For CVDs, mRNA therapy holds the potential for gene replacement strategies, where mRNA encoding for cardioprotective factors, such as VEGF or angiopoietins, can be introduced to enhance myocardial repair after ischemic injury (27). However, the challenge of ensuring sufficient mRNA stability and avoiding unwanted immune responses remains a critical consideration (28). Future research must critically assess the long-term efficacy and safety profiles of mRNA therapies in diverse CVDs contexts.

siRNA mediates gene silencing through the RNA interference (RNAi) pathway. siRNA can specifically target and degrade mRNA transcripts of genes implicated in CVDs, such as those involved in lipid metabolism or inflammatory pathways (21). Notably, the siRNA drug, Patisiran, which targets transthyretin mRNA, has shown success in treating hereditary transthyretin amyloidosis (29). While the precision of siRNA offers significant therapeutic prospects, challenges such as off-target effects, delivery methods, and the potential for immune responses need careful scrutiny (30). Comprehensive studies comparing the effectiveness of siRNA therapies across various CVDs models will elucidate their true therapeutic potential.

miRNA are short, non-coding RNA molecules that play a critical role in post-transcriptional regulation of gene expression (31). Dysregulation of specific miRNAs has been implicated in numerous cardiovascular disorders, including heart failure and atherosclerosis (32). The therapeutic use of miRNAs involves either inhibiting the activity of pathologically upregulated miRNAs or replacing downregulated protective miRNAs (15). For instance, let-7 family inhibition has demonstrated beneficial effects on cardiac remodeling following injury (16). Despite their potential, the use of miRNAs as therapeutics necessitates rigorous evaluation (33). The interplay of different miRNAs within cellular networks can lead to complex outcomes that warrant careful interpretation of preclinical findings, ensuring translational relevance to human pathology (33).

RNA aptamers are short, single-stranded RNA molecules that can bind to specific target proteins with high affinity and specificity (22). Their unique ability to inhibit protein functions offers therapeutic promise for diseases characterized by dysregulated protein interactions (22). For example, RNA aptamers targeting platelet-derived growth factor are being investigated for their role in inhibiting vascular smooth muscle cell proliferation, a key event in atherosclerosis (34). However, the clinical application of RNA aptamers is still in its infancy, requiring comprehensive evaluation of their pharmacodynamics, stability, and delivery mechanisms (35). Effective strategies to enhance their systemic availability and reduce renal clearance are pivotal to realizing their therapeutic potential (35).

### 2.2 Mechanisms of action

RNA therapeutics have emerged as a promising frontier in the treatment of CVDs. They offer precise and potent interventions by targeting specific molecular pathways involved in the pathogenesis of CVDs. RNA therapeutics exert their effects through distinct mechanisms depending on their type, providing targeted interventions for CVDs.

#### 2.2.1 siRNA mechanism

siRNA operates via the RNA interference (RNAi) pathway, a conserved biological process for sequence-specific gene silencing (30). Mechanistically, exogenous double-stranded siRNA is processed by the cytoplasmic enzyme Dicer into 21–23 nucleotide duplexes (21). One strand (the guide strand) is incorporated into the RNA-induced silencing complex (RISC), where it directs the complex to complementary mRNA sequences (30). Upon binding, the Argonaute 2 (AGO2) component of RISC cleaves the target mRNA, leading to its degradation and subsequent suppression of protein synthesis (25). For example, siRNA targeting PCSK9 binds to its mRNA with perfect complementarity, enabling RISC-mediated cleavage. This prevents PCSK9 protein synthesis, stabilizing hepatic LDL receptors and enhancing cholesterol clearance (36). The specificity of siRNA hinges on base-pairing fidelity, though off-target binding to partially complementary mRNAs remains a concern, necessitating rigorous bioinformatic design.

#### 2.2.2 mRNA mechanism

mRNA therapeutics function through a gene replacement strategy. Synthetic mRNA, engineered with modified nucleotides (e.g., pseudouridine) to evade immune detection, is delivered into the cytoplasm via LNPs (37). The mRNA bypasses nuclear entry and directly engages ribosomes for translation into functional proteins. For instance, VEGF-A mRNA is translated into vascular endothelial growth factor, which binds to endothelial cell receptors (VEGFR1/2), activating the PI3K-Akt and MAPK-ERK pathways to promote angiogenesis (38). Key challenges include optimizing codon usage for enhanced translational efficiency and incorporating untranslated regions to regulate stability (39). Recent advances in nucleoside modifications (e.g., 5-methoxyuridine) further prolong mRNA half-life by reducing RNase recognition (40).

#### 2.2.3 miRNA mechanism

miRNAs are endogenous, single-stranded RNAs that regulate gene expression post-transcriptionally (41). Therapeutic strategies involve either inhibiting overexpressed miRNAs or supplementing deficient miRNAs. miRNAs bind to partially complementary sequences in the 3′ untranslated region of target mRNAs via the miRNA-induced silencing complex (41). This interaction typically represses translation or recruits deadenylases and decapping enzymes to degrade the mRNA. For example, inhibition of the *let-7* family prevents its binding to pro-fibrotic transcripts (e.g., *TGF-βR1*), thereby attenuating cardiac fibrosis (42). Conversely, miR-21 mimics can suppress apoptotic pathways by targeting *PDCD4* and *PTEN* (43). The pleiotropic nature of miRNAs—each regulating hundreds of transcripts—requires careful validation to avoid unintended network-wide effects (41).

#### 2.2.4 RNA aptamer mechanism

RNA aptamers are structured, single-stranded RNAs that bind to specific proteins with high affinity through conformational complementarity (44). Their mechanism involves blocking active sites, disrupting protein-protein interactions, or altering protein conformation. For instance, aptamers targeting platelet-derived growth factor (PDGF) adopt a G-quadruplex structure that sterically hinders PDGF binding to its receptor, inhibiting vascular smooth muscle cell proliferation (45). Aptamers can also be engineered with chemical modifications (e.g., 2′-fluoro pyrimidines) to enhance nuclease resistance. Unlike antibodies, aptamers lack immunogenicity and can be thermally renatured, offering advantages in storage and delivery (46).

The diverse mechanisms of RNA therapeutics highlight their great potential in combating CVDs. As research progresses, these therapies are expected to bring more tailored and effective treatment options for patients, opening up new horizons in the field of cardiovascular medicine.

### 2.3 Advantages of RNA therapeutics in CVDs

RNA therapeutics represent a groundbreaking advancement in the treatment of CVDs, offering a variety of mechanisms to address the complexities associated with these conditions (19). One prominent advantage lies in their capacity for precise gene regulation (Table 1). Techniques such as siRNA and ASOs enable targeted silencing of specific genes implicated in the pathology of CVDs (47, 48). For example, studies have shown that targeting angiotensinogen via siRNA reduces blood pressure and mitigates cardiac hypertrophy in animal models, highlighting the potential of RNA-based approaches to manage hypertension (49). This selective targeting minimizes off-target effects and maximizes therapeutic efficiency, a significant improvement over conventional therapies, which often exhibit broader systemic effects and subsequent side effects (50).

**Table 1:** Advantages of RNA therapeutics in CVDs.

Advantages	Reasons	References
Targeted mechanism of action	RNA therapeutics, such as siRNA and mRNA, can be designed to specifically target genes implicated in CVDs. For example, siRNA targeting PCSK9 has been shown to reduce LDL cholesterol levels by degrading PCSK9 mRNA, enhancing LDL receptor availability on hepatocytes.	(36)
Reduced off-target effects	RNA-based therapies can be engineered to minimize off-target effects, leading to improved safety profiles. For instance, chemically modified siRNAs have been developed to reduce off-target gene silencing and immune activation, which is crucial for long-term applications in CVDs.	(30)
Potential for Long-lasting effects	Some RNA therapeutics, particularly those using long-lasting delivery systems, can provide sustained biological effects. For example, LNPs can deliver siRNA or mRNA to target tissues, resulting in prolonged gene silencing or protein expression. In CVDs, this approach can reduce the frequency of dosing and improve patient adherence (49–51).	(59–61)
Versatility	RNA therapeutics can be applied to a variety of CVDs, including hypercholesterolemia, heart failure, and vascular inflammation. For instance, siRNA targeting apolipoprotein B (mipomersen) has been used to treat homozygous familial hypercholesterolemia.	(63)
Rapid development and adaptation	The design and synthesis of RNA-based therapies can be quicker than traditional drug discovery processes. This allows for rapid adaptation in response to emerging CVDs or evolving patient profiles. For example, mRNA vaccines for COVID-19 were rapidly developed and deployed, showcasing the potential for RNA therapeutics to address urgent medical needs.	(10)
Personalized medicine	RNA-based approaches, when integrated with genomic data, enable personalized therapies. For example, genetic profiling can identify patients with specific mutations in target genes, allowing for the design of tailored antisense oligonucleotides (ASOs) to modulate these genetic anomalies. This approach has been demonstrated in the treatment of familial hypercholesterolemia (43).	(53)

Another advantage of RNA therapeutics is their flexibility in design and rapid development. Using advances in RNA synthesis and delivery systems, researchers can quickly adapt RNA-based therapies in response to emerging disease mechanisms or evolving patient profiles (Table 1) (51). Recent research has illustrated this adaptability; for instance, mRNA vaccines have been rapidly repurposed for addressing viral infections, showcasing a paradigm that can be applied to CVDs for delivering therapeutic factors, such as endothelial nitric oxide synthase (eNOS) mRNA to enhance endothelial function (52). The rapid translation of these technologies from laboratory research to clinical applications could provide timely and effective interventions for patients with acute cardiovascular conditions.

Moreover, RNA therapeutics foster the possibility of personalized medicine (53) (Table 1). The heterogeneity of CVDs necessitates the development of tailored therapies that consider individual genetic backgrounds, comorbidities, and specific disease presentations (54). RNA-based approaches, particularly when integrated with genomic data, enable clinicians to select optimal therapies for individual patients (55). For instance, genetic profiling to identify patients with specific mutations in target genes may allow for the design of personalized ASOs that effectively modulate these genetic anomalies (53). Such personalized strategies could enhance therapeutic outcomes, as exemplified by the clinical trials involving the ASO mipomersen, which demonstrated significant reductions in low-density lipoprotein (LDL) levels in patients with FH (56). However, while the promise of custom-tailored therapies is evident, challenges surrounding individual variability in response to RNA therapies and the ethical implications of genetic testing need to be carefully navigated (18).

Finally, RNA therapeutics exhibit a potential for sustained effects through innovative delivery methods, such as LNPs and exosomes, which can enhance the bioavailability and stability of RNA molecules in circulation (Table 1) (57). This approach not only extends the therapeutic window but could also reduce the frequency of dosing, improving patient adherence to treatment regimens (58). For example, studies have demonstrated that LNPs can effectively deliver RNA payloads to target tissues, resulting in sustained gene silencing over extended periods (59–61). However, while the initial results are encouraging, it is imperative to critically evaluate the long-term safety and efficacy of these delivery systems, as concerns regarding immunogenicity and potential systemic toxicity must be addressed in ongoing and future trials (62).

## 3 RNA therapeutics in CVDs

**Figure 2:**
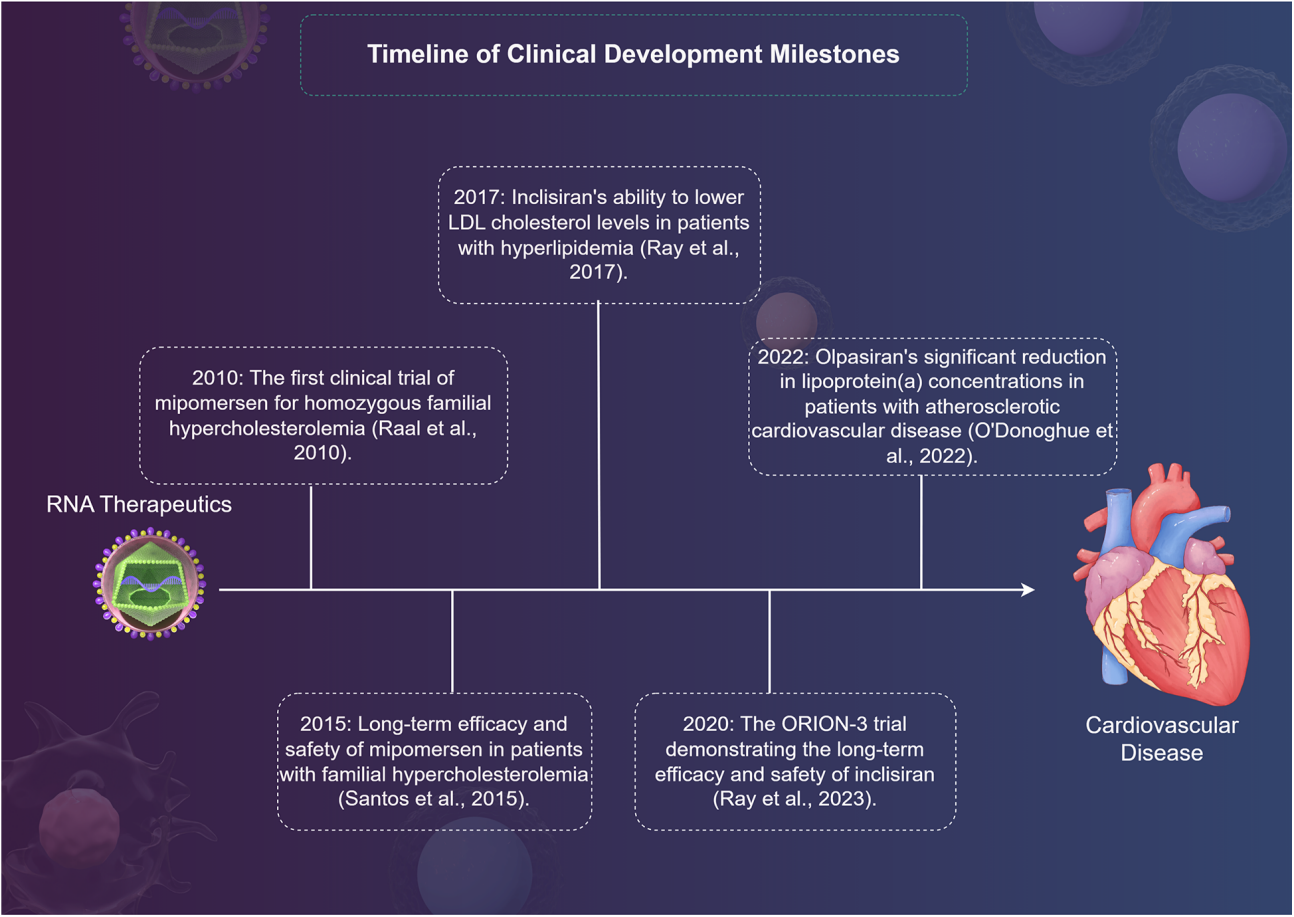
A timeline of clinical development milestones for RNA therapy in cardiovascular disease by Figdraw.

RNA therapeutics have emerged as a promising strategy in the management of various CVDs leveraging their ability to modulate gene expression at the transcriptional or translational level. The clinical research landscape surrounding these therapies has rapidly evolved, showcasing their potential in targeting lipid metabolism, improving cardiac function, and addressing a range of genetic disorders (Table 2). Key milestones in the clinical development of RNA therapeutics include the first clinical trial of mipomersen for homozygous familial hypercholesterolemia (FH) in 2010, which demonstrated its potential to lower LDL cholesterol levels (63). Subsequently, in 2015, long-term data from mipomersen treatment in patients with FH confirmed its sustained efficacy and safety profile (64). In 2017, inclisiran, an siRNA therapeutic targeting PCSK9, emerged as a promising agent for lipid management, showing significant reductions in LDL cholesterol levels in patients at high cardiovascular risk (65). Building on these findings, the ORION-3 trial in 2020 provided further evidence of inclisiran's long-term efficacy and safety in reducing LDL cholesterol (66). Most recently, in 2022, olpasiran, an siRNA targeting lipoprotein(a), demonstrated a significant reduction in Lp(a) levels in patients with atherosclerotic cardiovascular disease, marking a potential breakthrough in managing this challenging risk factor (67) (Figure 2).

**Table 2:** Clinical studies on the application of RNA therapy in the treatment of CVDs.

Name	Target	Type of RNA	Strategy (inhibition/overexpression)	Condition	Type of comparator	Phase	NCT number	Clinical efficacy	References
VEGF-A	VEGFR	mRNA	Overexpression	Heart failure	—	1	NCT03370887	VEGF-A mRNA may have therapeutic potential for regenerative angiogenesis.	Gan et al. (13)
VEGF-A	VEGFR	mRNA	Overexpression	Vascular and inflammatory conditions	—	2	NCT03370887	VEGF-A promotes therapeutic angiogenesis, aiming to improve outcomes in patients with coronary artery disease through enhanced blood vessel growth and myocardial regeneration.	Anttila et al. (14)
Mipomersen	Apolipoprotein B	ASO	Inhibition	Homozygous familial hypercholesterolaemia	Placebo	3	NCT00607373	Mipomersen significantly lowered LDL cholesterol concentrations compared to a placebo	Raal et al. (63)
Mipomersen	Apolipoprotein B	ASO	Inhibition	Familial hypercholesterolaemia	Placebo	3	NCT00694109	Mipomersen treatment for 104 weeks reduced atherosclerotic lipoproteins and showed comparable safety.	Santos et al. (64)
Inclisiran	PCSK9	siRNA	Inhibition	Hyperlipidemia with increased LDL	Placebo	2	NCT02597127	In patients at high cardiovascular risk with elevated LDL cholesterol levels, inclisiran can lower PCSK9 and LDL cholesterol levels.	Ray et al. (65)
Inclisiran	PCSK9	siRNA	Inhibition	Atherosclerotic CVDs	Placebo	2	NCT03060577	Twice-yearly inclisiran provided sustained reductions in LDL cholesterol and PCSK9 concentrations.	Ray et al. (66)
Olpasiran	Lipoprotein(a)	siRNA	Inhibition	Atherosclerotic CVDs	Placebo	2	NCT04270760	Olpasiran therapy significantly reduced lipoprotein(a) concentrations in patients with established atherosclerotic CVDs.	O’Donoghue et al. (67)
Zilebesiran	Angiotensinogen	siRNA	Inhibition	Hypertension	Placebo	2	NCT05127272	Zilebesiran significantly reduced blood pressure in patients with mild to moderate hypertension.	Desai et al. (69)
Zilebesiran	Angiotensinogen	siRNA	Inhibition	Hypertension	Placebo	2	NCT05324488	Zilebesiran as add-on therapy significantly reduced blood pressure in patients with inadequately controlled hypertension.	Saxena et al. (70)
AKCEA-APOCIII-LRx	apoC-III protein	mRNA	Overexpression	Hypertriglyceridaemia	Placebo	1/2a	NCT02900027	Treatment of hypertriglyceridaemic subjects with AKCEA-APOCIII-LRx results in a broad improvement in the atherogenic lipid profile.	Alexander et al. (72)
Olpasiran	Lipoprotein(a)	siRNA	Inhibition	Atherosclerotic CVDs	Placebo	1	NCT03626662	Olpasiran can effectively reduce Lp(a) in individuals with elevated plasma Lp(a) concentrations.	Koren et al. (73)
Serelaxin	Relaxin-2	mRNA	Overexpression	Heart failure	Placebo	3	NCT01870778	Serelaxin has a potentially beneficial effect on cardiovascular mortality and exacerbation of heart failure in patients with acute heart failure.	Teerlink et al. (74)
CDR132l	miR-132	ASO	Inhibition	Heart failure	Placebo	1	NCT04045405	CDR132l is a safe and well-tolerated antisense drug that shows encouraging potential for improving cardiac function in heart failure patients.	Täubel et al. (75)
CDR132l	miR-132	siRNA	Inhibition	Heart failure	Placebo	2	NCT05350969	CDR132l can prevent or reverse cardiac remodeling in heart failure after myocardial infarction.	Bauersachs et al. (76)
ARO-ANG3	ANGPTL3	siRNA	Inhibition	Vascular and inflammatory conditions	Placebo	—	NCT03626662	ARO—ANG3 can reduce LDL-C and triglyceride rich lipoproteins, the mixed dyslipidemia in patients with atherosclerosis sex plays a protective role.	Watts et al. (77)
Inclisiran	PCSK9	siRNA	Inhibition	Atherosclerotic CVDs (ASCVDs)	—	2	NCT02597127	Inclisiran significantly reduces apolipoprotein B, non-HDL cholesterol, and lipoprotein (a).	Leiter et al. (78)
inclisiran	PCSK9	siRNA	Inhibition	Atherosclerotic CVDs	Placebo	3	NCT03060577	Inclisiran, added to maximally tolerated lipid-lowering therapy, is effective, safe, and significantly reduces LDL-C in high cardiovascular risk patients.	Raal et al. (79)

CVDs, cardiovascular diseases; LDL, low-density lipoprotein; PCSK9, proprotein convertase subtilisin/kexin Type 9; LDL-C, low-density lipoprotein cholesterol; Lp(a)-C, lipoprotein(a) cholesterol; VEGF-A, vascular endothelial growth factor-A.

### 3.1 Hypertension

Hypertension, a major risk factor for cardiovascular diseases (CVDs), has emerged as a promising target for RNA therapeutics. Recent studies have shown that RNA interference (RNAi) can be used to silence genes involved in blood pressure regulation, offering a novel approach to hypertension management. RNAi-based therapies target specific genes involved in the renin-angiotensin system, a key pathway in blood pressure regulation. For example, small interfering RNA (siRNA) targeting angiotensinogen, a key component of the RAS, has demonstrated significant blood pressure-lowering effects in preclinical models (68). By silencing the angiotensinogen gene, RNAi therapies can reduce the production of angiotensin II, a potent vasoconstrictor, thereby lowering blood pressure.

Recent clinical trials have shown promising results. For instance, zilebesiran, an siRNA therapeutic targeting angiotensinogen, has been evaluated in the KARDIA-1 trial, demonstrating significant blood pressure reductions in patients with mild to moderate hypertension (69). The KARDIA-2 trial further confirmed the efficacy and safety of zilebesiran as an add-on therapy in patients with inadequately controlled hypertension (70). These studies highlight the potential of RNAi-based therapies to provide long-lasting blood pressure control with minimal side effects.

The development of RNA therapeutics for hypertension is still in its early stages, but the initial results are encouraging. Future research should focus on optimizing delivery methods to enhance the efficiency and safety of RNAi therapies. Additionally, long-term studies are needed to evaluate the sustained effects and potential side effects of these treatments in diverse patient populations.

### 3.2 Hypercholesterolemia

Hypercholesterolemia, particularly FH, has been a primary target for RNA-based therapies due to the associated cardiovascular risks. Mipomersen, an antisense oligonucleotide targeting apolipoprotein B, has demonstrated significant efficacy in reducing LDL cholesterol levels in patients with homozygous FH (63). It was approved by the U.S. Food and Drug Administration (FDA) in 2012 for this indication. However, the European Medicines Agency (EMA) rejected its marketing authorization application in 2013 due to concerns regarding its safety profile and the clinical benefit-risk ratio (64). Despite the approval in the US, ongoing monitoring of its long-term safety and efficacy is essential, as highlighted by the results from the open-label extension study (64). In a long-term study by Santos et al. (64), mipomersen treatment for 104 weeks reduced atherosclerotic lipoproteins and showed comparable safety. Despite these findings, the study's limitations include a relatively small sample size and potential selection bias, which may affect the generalizability of the results. While studies highlight the effectiveness of mipomersen in lipid lowering efficacy, particularly in different genetic backgrounds, warrant critical evaluation. For instance, while mipomersen's lipoprotein effects are well-documented, real-world effectiveness in diverse populations may vary (71). These differences underscore the need for careful patient selection and continuous monitoring in clinical practice.

Inclisiran, a siRNA that targets proprotein convertase subtilisin/kexin Type 9(PCSK9), shows significant promise as an effective therapy for lowering LDL cholesterol levels, particularly in patients with FH. The ORION-4 trial illustrated inclisiran's ability to maintain lower cholesterol levels through biannual dosing, emphasizing its ease of administration and potential for long-term adherence (65). Additionally, long-term efficacy reported in the open-label extension 3 (ORION-3) reinforces inclisiran's potential for sustained lipid management in high-risk populations (66). However, the trial's limitations include the potential for bias due to the open-label extension phase, which may influence patient and physician behavior. Additionally, long-term safety data are still limited, highlighting the need for further longitudinal studies to fully assess its safety profile.

Moreover, the introduction of volanesorsen, a novel antisense drug targeting apolipoprotein C-III (APOC3), has gained attention. A study revealed its ability to significantly lower triglycerides and atherogenic lipoproteins, positioning it as a valuable addition to the therapeutic arsenal against CVDs exacerbated by hypertriglyceridemia (72). It was approved by the EMA in 2018 for the treatment of patients with homozygous familial chylomicronemia syndrome (72). Conversely, it was rejected by the FDA in 2019 due to concerns regarding its safety and efficacy, particularly in relation to injection-site reactions and neurological adverse events (72). This discrepancy in regulatory decisions highlights the ongoing debate about the risk-benefit profile of volanesorsen and the need for further research to address these concerns.

### 3.3 Lipoprotein(a) management

Lipoprotein(a) [Lp(a)] is increasingly recognized as a significant independent risk factor for cardiovascular events. Disruption of Lp(a) synthesis presents a novel therapeutic avenue. A study exploring olpasiran, a siRNA that reduces Lp(a) levels, exhibited promising outcomes in lowering Lp(a) cholesterol and correcting LDL cholesterol levels (67). O'Donoghue et al. (67) reported that olpasiran significantly reduced Lp(a) cholesterol levels. However, the study's limitations include a short follow-up period, which may not fully capture long-term efficacy and safety outcomes. Furthermore, the trial's population was predominantly Caucasian, raising questions about the generalizability of the results to diverse populations. Koren et al. (73) also demonstrated the effective preclinical and clinical development of siRNA-targeting strategies against Lp(a), reinforcing the therapeutic prospect of RNA molecules in mitigating cardiovascular risks tied to elevated Lp(a) levels. These studies depict a burgeoning field that targets previously hard-to-manage lipid fractions, indicating the promising trajectory of RNA therapeutics in CVDs.

Despite these advancements, variability exists in the response to Lp(a) lowering therapies across studies and populations. Factors such as genetic polymorphisms and concurrent metabolic conditions can influence individual responses to RNA therapeutics. Hence, critically assessing patient backgrounds and potential biomarkers prior to treatment initiation remains essential.

### 3.4 Heart failure

Heart failure remains an area burdened by high morbidity and mortality rates. RNA-based therapies hold promise in addressing underlying mechanisms of cardiac dysfunction. Serelaxin, a recombinant form of relaxin acquired via mRNA technology, was evaluated in patients with acute heart failure. Teerlink et al. (74) verified that serelaxin treatment contributed positively to heart failure outcomes by improving clinical symptoms and inflammatory parameters. However, the study's limitations include a relatively small sample size and the lack of a long-term follow-up period to assess sustained benefits. Additionally, Gan et al. (13) showcased the potential of intradermal delivery of modified mRNA encoding VEGF-A, which promoted angiogenesis in diabetic patients undergoing procedures like coronary artery bypass grafting. Here, RNA therapeutics not only treat existing conditions but may also foster regenerative capabilities in cardiovascular tissues.

One of the pioneering studies involved targeting microRNA-132, which is associated with cardiac hypertrophy and remodeling. Täubel et al. (75) demonstrated that antisense therapy against microRNA-132 was safe and well-tolerated without significant adverse effects, indicating its potential as a novel therapeutic strategy in HF. Additionally, the application of CDR132l has been evaluated in patients following myocardial infarction with reduced ejection fraction (76). The HF-REVERT trial aims to determine its efficacy in improving functional outcomes and cardiac remodeling. Initial results appear promising, yet long-term effects and comprehensive safety profiles are still under investigation. The trial's limitations include the potential for bias due to the single-center design and the need for larger, multicenter studies to confirm the findings.

### 3.5 Vascular and inflammatory conditions

Emerging RNA therapies, such as those targeting angiopoietin-like proteins, have demonstrated capabilities in altering lipid and lipoprotein profiles in relevant inflammatory conditions (77). Therapies targeting angiopoietin-like protein 3 (ANGPTL3) with arcturus RNAi oligonucleotide-angiopoietin-like protein 3(ARO-ANG3) present a novel approach in managing abnormal lipid levels, although more extensive trials are warranted to illustrate disease-modifying effects across broader patient demographics (77). Moreover, therapeutic angiogenesis may enhance outcomes for coronary artery disease patients undergoing surgery (14). The ongoing EPICCURE trial investigates the safety of AZD8601, a vascular endothelial growth factor-A165 (VEGF-A165) mRNA, administered via epicardial injections during revascularization. The study aims to evaluate safety and exploratory efficacy through various imaging and assessment methods (14). The ongoing exploration of RNA strategies underscores their potential across various pathophysiological processes tied to CVDs.

The landscape of RNA therapeutics for CVDs management reflects significant promise, with clinical trials providing essential insights into their efficacy and safety (19). The variety of approaches leveraging RNA technology illustrates its adaptability and innovation in targeting specific disease mechanisms (9). Nonetheless, consistency in outcomes, long-term safety, and comparative effectiveness with existing therapies must guide future research. A comprehensive understanding of patient-specific factors will be essential in optimizing these advanced therapeutic modalities and achieving better clinical outcomes in CVDs. As the field continues to evolve, thorough evaluation of ongoing and future studies will be essential in establishing RNA therapeutics as a mainstay in CVDs management.

### 3.6 Thrombosis and fibrinolysis

Thrombosis and fibrinolysis play crucial roles in the pathogenesis of CVDs, particularly in conditions such as myocardial infarction and stroke. Targeting these pathways offers a promising avenue for therapeutic intervention. RNA therapeutics have shown potential in modulating thrombosis and fibrinolysis through various mechanisms.While the research on RNA therapeutics targeting thrombosis and fibrinolysis is still in its early stages, several preclinical studies have demonstrated their potential. For example, a preclinical study of an siRNA therapeutic targeting TF showed promising results in terms of safety and efficacy (68). Future research should focus on optimizing delivery methods, improving stability, and evaluating long-term safety and efficacy in larger clinical trials.

## 4 Safety and efficacy of RNA therapeutics

RNA therapeutics have shown varying success in clinical trials for cardiovascular conditions, with some trials achieving significant improvements in patient outcomes, while others have revealed limitations in efficacy (18). Safety concerns arise, particularly regarding off-target effects and immune responses. Understanding these factors is crucial for optimizing treatment protocols and ensuring the long-term success of RNA-based therapies.

### 4.1 Overview of clinical trial results

Clinical trials evaluating RNA therapeutics for CVDs have demonstrated both promise and variability in outcomes. For instance, inclisiran, an siRNA targeting PCSK9, achieved sustained LDL cholesterol reductions with biannual dosing in the ORION-4 trial, highlighting its potential for long-term lipid management (65). Conversely, early trials of mRNA encoding SERCA2a for heart failure showed transient improvements in left ventricular function but failed to demonstrate durable benefits in larger cohorts, underscoring challenges in achieving consistent therapeutic effects (80, 81). Similarly, while siRNA-based therapies like zilebesiran (targeting angiotensinogen) significantly lowered blood pressure in hypertensive patients (69, 70), trials of miRNA-132 inhibitors (e.g., CDR132l) reported mixed results, with modest effects on cardiac remodeling (75, 76). These discrepancies emphasize the need to address variability in patient selection, dosing regimens, and delivery efficiency. Recent meta-analyses further suggest that RNA therapeutics exhibit higher efficacy in genetically defined subgroups (e.g., familial hypercholesterolemia) compared to heterogeneous populations, necessitating precision medicine approaches (82).

### 4.2 Safety profiles of different RNA modalities

#### 4.2.1 Off-target effects

One of the critical safety concerns associated with RNA therapeutics is the potential for off-target effects (83). RNA molecules, especially siRNAs and miRNAs, can interact with unintended mRNAs, leading to unanticipated biological consequences (84). For example, the administration of siRNAs can inadvertently downregulate essential genes, which can result in detrimental effects (85). For example, early siRNA candidates targeting apolipoprotein B (mipomersen) were associated with hepatic steatosis, likely due to off-target suppression of lipid metabolism genes (63, 71). Similarly, miR-122 inhibitors, while effective in reducing hepatitis C viral load, inadvertently disrupted cholesterol homeostasis, leading to dyslipidemia (86). Advances in bioinformatic design tools (e.g., machine learning algorithms for predicting siRNA specificity) and chemical modifications (e.g., 2′-O-methylation) have mitigated these risks, as evidenced by newer siRNA therapies like olpasiran, which selectively lowers lipoprotein(a) without significant off-target activity (67, 73).

#### 4.2.2 Immune responses

Another significant safety concern is the immune response to RNA therapeutics (87). Unmodified RNA has the potential to stimulate innate immune pathways, leading to inflammatory responses (88). Studies have shown that RNA-based therapies can trigger cytokine storms and other immune reactions, prompting a need for the development of modified RNA formats that minimize immunogenicity (89, 90). Recent advancements have introduced chemical modifications to mRNA to improve stability and reduce immune recognition (91). Modified nucleotides, such as 5-methylcytidine or 2ʹ-O-methylation, have been shown to dampen immune activation while enhancing translation efficiency (92). These modifications have achieved promising outcomes in several preclinical settings but necessitate careful monitoring in human studies to fully evaluate safety (93).

#### 4.2.3 Organ-specific toxicity

Accumulation of RNA therapeutics in non-target tissues remains a concern. For example, LNPs preferentially localize to the liver but may deposit in the spleen or kidneys, raising risks of immune cell activation or renal toxicity (94). Preclinical studies of VEGF-A mRNA revealed edema in non-cardiac tissues due to systemic leakage, necessitating optimized delivery routes (e.g., localized epicardial injections in the EPICCURE trial) (14). Viral vectors, such as AAVs, pose risks of hepatotoxicity and neutralizing antibody formation, limiting their re-dosing potentia (95).

#### 4.2.4 Long-term safety

Longitudinal data remain sparse. While mipomersen showed acceptable safety over 104 weeks in familial hypercholesterolemia (64), volanesorsen (targeting APOC3) was linked to thrombocytopenia and injection-site reactions, prompting FDA rejection despite EMA approval (72). Emerging platforms, such as exosome-mediated delivery, offer improved biocompatibility but require rigorous evaluation of biodistribution and chronic toxicity (96).

## 5 Advancements in RNA delivery systems for cardiovascular therapeutics

The effective use of RNA therapeutics for CVDs significantly depends on the performance of delivery systems (19). The distinct challenges posed by RNA molecules, including their natural instability, vulnerability to degradation, and relatively large size, require innovative strategies to guarantee their proper uptake in targeted tissues (18).

### 5.1 Novel delivery systems

#### 5.1.1 Viral vectors

**Figure 3:**
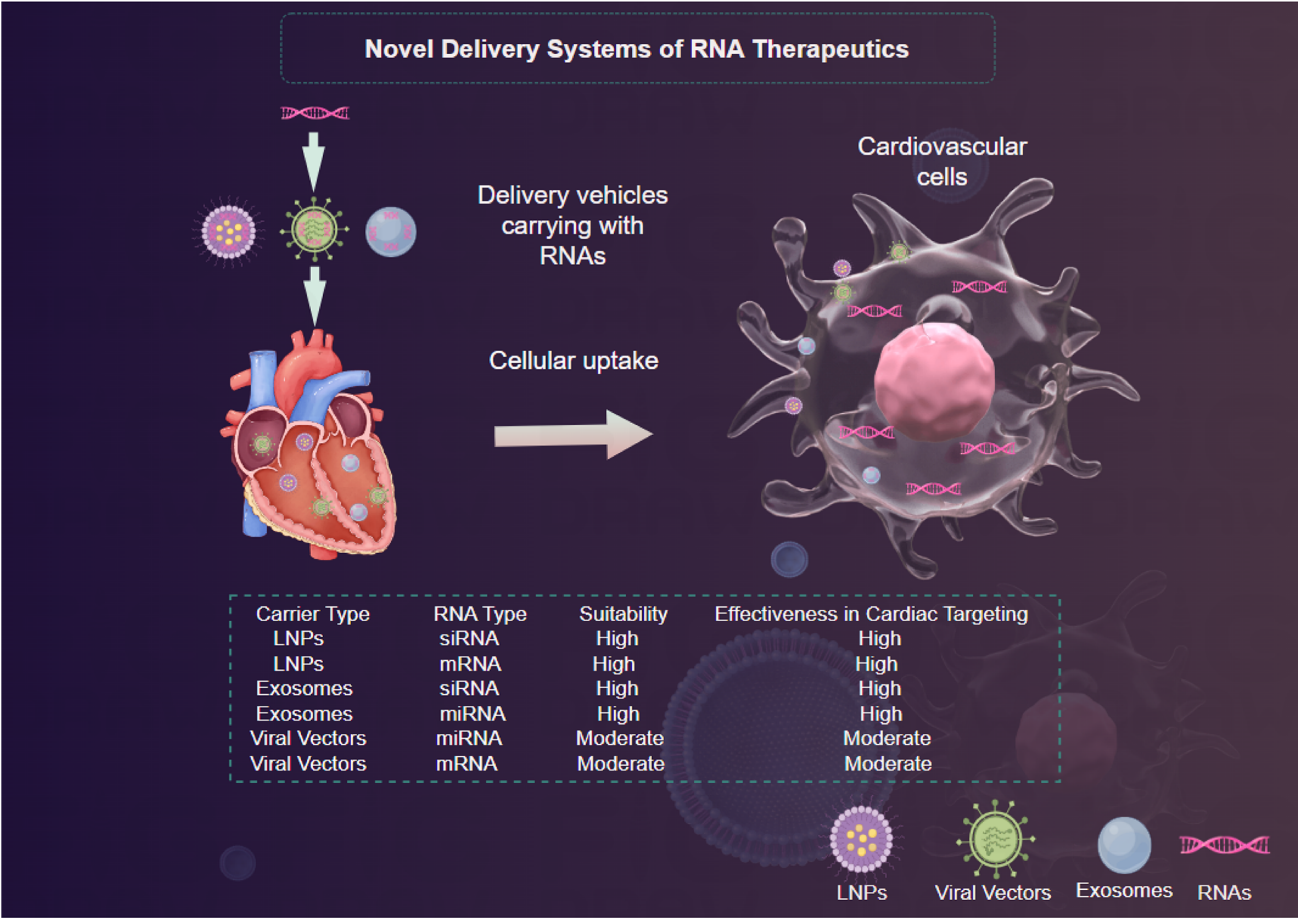
Novel delivery systems of RNA therapeutics demonstrated by Figdraw.

Viral vectors have long been utilized for gene therapy; however, their application in RNA therapeutics is gaining traction (97). Adeno-associated viruses (AAVs) and lentiviral vectors have shown promise due to their ability to facilitate sustained expression of the therapeutic RNA after delivery (88). These vectors can be engineered to exhibit tissue tropism, enhancing their delivery to specific cell types in the cardiovascular system (88) (Figure 3). Nevertheless, concerns regarding safety, such as immunogenic responses and insertional mutagenesis, limit their clinical applicability (95). The recent advent of less immunogenic variants of AAVs and the use of transient expression vectors may help mitigate such risks while advancing the therapeutic potential of RNA interventions (88).

#### 5.1.2 Lipid nanoparticles

Lipid nanoparticles (LNPs) have emerged as one of the most promising platforms for RNA delivery due to their ability to encapsulate RNA molecules, enhance cellular uptake, and facilitate endosomal escape (94) (Figure 3). LNPs can be engineered to improve their pharmacokinetic properties and target specific tissues through modifications that alter surface charge, lipid composition, and size (98). Recent studies demonstrate that LNPs containing mRNA encoding proteins such as SERCA2a successfully ameliorate heart function in animal models of heart failure (99, 100). The versatility and scalability of LNPs systems make them a frontrunner in RNA delivery, however, ongoing challenges such as potential immunogenicity and stability during storage must be addressed (101).

#### 5.1.3 Exosomes

Exosomes, small extracellular vesicles secreted by various cell types, have emerged as innovative delivery vehicles thanks to their natural ability to facilitate intercellular communication (96). Exosomes can encapsulate RNA molecules, providing inherent biocompatibility and reducing the likelihood of immune rejection (96) (Figure 3). Furthermore, their lipophilic membranes enhance their ability to penetrate target cells (96). Research has indicated that exosome-mediated delivery of miRNAs can significantly modulate pathological processes in CVDs, with promising results in preclinical models of atherosclerosis and heart failure (102, 103). However, optimizing the isolation, characterization, and loading of therapeutic RNA into exosomes remains an ongoing challenge that necessitates further investigation (104).

### 5.2 Innovations in delivery methods enhancing cardiovascular targeting

A multitude of recent innovations are focused on improving the targeted delivery of RNA therapeutics to the cardiovascular system (19). These include the development of ligand-targeted delivery systems, use of biodegradable polymers, and advancement in needle-free injection systems (19).

Ligand-targeted delivery leverages specific ligands that bind selectively to receptors overexpressed on cardiac or vascular cells (105). For instance, the conjugation of RNA constructs to antibodies or peptides that target inflammation markers has demonstrated enhanced uptake in diseased tissues (62). Moreover, biodegradable polymers represent a promising strategy for controlled and sustained release of RNA therapeutics (93). Such systems protect RNA from degradation while facilitating gradual release, enhancing therapeutic efficacy and minimizing the need for repetitive dosing (106). Finally, advancements in needle-free injection systems have improved the feasibility of delivering RNA therapeutics via intradermal or transdermal routes, promoting patient compliance (107). These innovations have the potential to simplify the delivery process while achieving greater concentration at the target site.

### 5.3 Comparative analysis and challenges of delivery methods

To provide a clear comparison of the various RNA delivery methods discussed, the following Table 3 summarizes their key features. This comparative analysis highlights the unique features and potential applications of each delivery method in the context of RNA therapeutics for CVDs. While LNPs and exosome-mediated delivery offer significant advantages in terms of biocompatibility and targeting potential, ongoing research is focused on addressing their respective challenges to optimize their performance in clinical settings.

**Table 3:** Comparative analysis of delivery methods.

Delivery method	Composition	Advantages	Challenges	Recent advances
LNPs	Ionizable lipids, phospholipids, cholesterol, PEG-lipids	Enhanced stability, efficient cellular uptake, scalability	Immunogenicity, off-target effects	Novel ionizable lipids, targeting ligands
Exosome-mediated delivery	Lipid bilayer membrane, proteins, RNA	Biocompatibility, enhanced cellular uptake, targeting potential	Isolation and characterization, scalability	Engineered exosomes, targeting ligands
Viral vectors	Viral capsid proteins, genetic material	Sustained expression, tissue tropism	Immunogenicity, insertional mutagenesis	Less immunogenic variants, transient expression
Ligand-targeted delivery	Ligands conjugated to nanoparticles	Enhanced targeting, reduced off-target effects	Ligand stability, manufacturing complexity	Novel ligands, multi-ligand systems
Biodegradable polymers	Biodegradable polymer matrices	Controlled release, reduced immunogenicity	Polymer degradation byproducts, loading efficiency	Advanced polymer formulations, hybrid systems

The delivery of RNA therapeutics faces several critical challenges. First, RNA is prone to degradation by ribonucleases present in blood and tissues, which necessitates protective formulations to enhance its stability (105). Additionally, the hydrophilic nature of RNA molecules creates barriers to cellular uptake, limiting their bioavailability in the target tissues (105). Furthermore, the desired specificity of RNA delivery remains a significant hurdle. Effective targeting requires distinguishing between healthy tissues and sites of pathological relevance, such as ischemic myocardium or atherosclerotic plaques (108). Achieving this specificity minimizes off-target effects, which can lead to unwanted systemic consequences (108). Current delivery methods often struggle with these issues, highlighting the urgent need for refined strategies that can deliver RNA therapeutics efficiently and safely to the cardiovascular system.

## 6 Emerging strategies and future directions

The field of CVDs management is undergoing rapid transformation, with RNA therapeutics leading the way in innovative approaches (105). Emerging strategies are centered on personalized medicine, and the roles of artificial intelligence (AI) and bioinformatics, as well as anticipated future directions in clinical applications and ongoing research.

Emerging RNA therapies, such as those targeting angiopoietin-like proteins, have demonstrated capabilities in altering lipid and lipoprotein profiles in relevant inflammatory conditions (77). Therapies targeting ANGPTL3 with ARO-ANG3 present a novel approach in managing abnormal lipid levels, although more extensive trials are warranted to illustrate disease-modifying effects across broader patient demographics (77). A recent review comprehensively discusses several potential RNA therapeutics under development, emphasizing the diversity of targets and mechanisms being explored (109). These include novel siRNA and mRNA approaches aimed at modulating key pathways in vascular inflammation and atherosclerosis. The ongoing EPICCURE trial, investigating the safety of AZD8601 (a VEGF-A165 mRNA administered via epicardial injections during revascularization), exemplifies the translational potential of RNA strategies in improving outcomes for coronary artery disease patients (14). The integration of these innovative therapies underscores the dynamic landscape of RNA therapeutics in addressing unmet clinical needs in cardiovascular medicine.

Personalized medicine, which tailors treatment based on individual patient characteristics, is increasingly feasible with RNA technologies (53). Genomic profiling allows for the identification of specific genetic mutations and expression profiles that can be targeted effectively with RNA therapeutics (110). For instance, the stratification of patients based on the expression levels of relevant miRNAs has shown promise in predicting responses to treatments (111). Recent studies have demonstrated that employing patient-specific RNA profiles can guide bespoke therapies, such as tailored cancer immunotherapies (112), personalized gene editing for genetic disorders (113), and customized RNA vaccines for infectious diseases (114). However, the integration of personalized approaches remains challenging due to inconsistencies in RNA expression across populations and the complexity of biomarker identification (82). Hence, rigorous validation of RNA signatures in diverse cohorts is essential to optimize personalized treatment strategies and establish clinical relevance (82).

The integration of AI and bioinformatics into RNA therapeutic development is an exciting frontier with the potential to enhance numerous aspects of research and clinical application (115). AI algorithms can analyze large-scale genomic and transcriptomic datasets, identifying patterns and predicting responses to RNA therapeutics (84). For example, machine learning models have been used to discover novel miRNA targets and predict their interactions with specific mRNAs, accelerating the discovery of potential therapeutic candidates (116). Bioinformatics tools are equally crucial for understanding the complex regulatory networks governing RNA dynamics and their implications in disease processes (117). Nevertheless, challenges remain in validating AI predictions within biological contexts (118). A critical evaluation of AI-generated hypotheses through experimental validation is necessary to ensure their translational relevance and applicability in clinical settings (119). Establishing collaborative frameworks between computational scientists and cardiovascular researchers will be vital to leverage the full potential of these technologies (119).

Looking ahead, the future of RNA therapeutics in CVDs management appears promising yet complex (62). Future research must prioritize addressing the key challenges in delivery mechanisms, safety, and efficacy discussed in previous sections. The development of novel delivery vehicles that enhance tissue-targeting and minimize immune responses is critical for the successful translation of RNA-based therapies from bench to bedside. Additionally, continued efforts should focus on the refinement of regulatory frameworks to accommodate the unique challenges posed by RNA therapies, ensuring a robust pathway for clinical approval while maintaining patient safety (20). As the field evolves, the collaboration between academic, governmental, and industry stakeholders will be crucial in fostering an environment conducive to innovation. Finally, larger-scale clinical trials are warranted to assess the long-term efficacy and safety profiles of RNA therapeutics in diverse patient populations. Emphasis should also be placed on conducting comparative effectiveness research to ascertain the relative benefits of RNA modalities against existing therapies, thus providing clearer guidance for clinicians in decision-making processes.

circRNAs have emerged as promising biomarkers and therapeutic agents in various diseases, including CVDs. These non-coding RNAs are characterized by their covalently closed loop structures, which make them highly stable in biological samples. In CVDs, circRNAs have been identified as potential biomarkers and therapeutic targets. For instance, circRNA_0001946 has been shown to be significantly upregulated in patients with coronary artery disease and can be used as a potential biomarker for early diagnosis (120). Additionally, circRNA_000911 has been identified as a regulator of vascular smooth muscle cell proliferation and could serve as a therapeutic target for atherosclerosis (121). The integration of circRNA studies into RNA therapeutic research could provide new insights into the mechanisms of CVDs and offer novel therapeutic strategies. Future research should focus on further exploring the roles of circRNAs in CVDs and developing circRNA-based therapies to improve patient outcomes.

While personalized RNA therapies hold immense potential for tailored treatment, they also raise several ethical concerns that warrant careful consideration. One primary issue is the risk of genetic discrimination. As these therapies rely on genomic profiling, there is a possibility that patients' genetic information could be misused, leading to discrimination in employment or insurance (110). Ensuring patient confidentiality and establishing robust data protection policies are crucial to mitigate this risk. Another ethical challenge is the question of informed consent. Patients must be fully informed about the potential benefits and risks of personalized RNA therapies, including the uncertainties associated with long-term safety and efficacy (111). Efforts to make these therapies more affordable and accessible are necessary to ensure fair distribution of healthcare resources. Addressing these ethical issues is vital to the successful implementation of personalized RNA therapies in clinical practice. Future research should focus not only on the scientific and technical aspects but also on the development of ethical guidelines and policies to govern the use of these innovative treatments.

## 7 Conclusion

In summary, this review has demonstrated that RNA therapeutics represent a promising frontier in the management of CVDs, showing potential in personalized treatment approaches and combination therapies. The evidence highlights the significant advancements in RNA modalities, despite challenges such as safety profiles and delivery mechanisms. As these innovations evolve, continued research and collaboration across disciplines are essential to optimize therapeutic strategies and establish robust clinical applications. By fostering interdisciplinary partnerships, we can accelerate the translation of RNA-based interventions into clinical practice, ultimately improving patient outcomes in cardiovascular care.
